# Fecal microbiota as a predictor of acupuncture responses in patients with postpartum depressive disorder

**DOI:** 10.3389/fcimb.2023.1228940

**Published:** 2023-11-20

**Authors:** Yu-Mei Zhou, Jin-Jun Yuan, Yu-Qin Xu, Yan-Hua Gou, Yannas Y. X. Zhu, Chen Chen, Xing-Xian Huang, Xiao-Ming Ma, Min- Pi, Zhuo-Xin Yang

**Affiliations:** ^1^ Department of Acupuncture, The Fourth Clinical Medical College of Guangzhou University of Chinese Medicine, Shenzhen, Guangdong, China; ^2^ Department of Acupuncture and Tuina, Shenzhen Maternal and Child Health Care Hospital, Shenzhen, China

**Keywords:** fecal microbiota, postpartum depressive disorder, acupuncture, the gut-brain axis, predictive biomarker, *Paraprevotella*, *Desulfovibrio*

## Abstract

**Background:**

There are several clinical and molecular predictors of responses to antidepressant therapy. However, these markers are either too subjective or complex for clinical use. The gut microbiota could provide an easily accessible set of biomarkers to predict therapeutic efficacy, but its value in predicting therapy responses to acupuncture in patients with depression is unknown. Here we analyzed the predictive value of the gut microbiota in patients with postpartum depressive disorder (PPD) treated with acupuncture.

**Methods:**

Seventy-nine PPD patients were enrolled: 55 were treated with acupuncture and 24 did not received any treatment. The 17-item Hamilton depression rating scale (HAMD-17) was used to assess patients at baseline and after eight weeks. Patients receiving acupuncture treatment were divided into an acupuncture-responsive group or non-responsive group according to HAMD-17 scores changes. Baseline fecal samples were obtained from the patients receiving acupuncture and were analyzed by high-throughput 16S ribosomal RNA sequencing to characterize the gut microbiome.

**Results:**

47.27% patients responded to acupuncture treatment and 12.5% patients with no treatment recovered after 8-week follow-up. There was no significant difference in α-diversity between responders and non-responders. The β-diversity of non-responders was significantly higher than responders. *Paraprevotella* and *Desulfovibrio* spp. were significantly enriched in acupuncture responders, and these organisms had an area under the curve of 0.76 and 0.66 for predicting responder patients, respectively.

**Conclusions:**

*Paraprevotella* and *Desulfovibrio*are may be useful predictive biomarkers to predict PPD patients likely to respond to acupuncture. Larger studies and validation in independent cohorts are now needed to validate our findings.

## Graphical Abstract

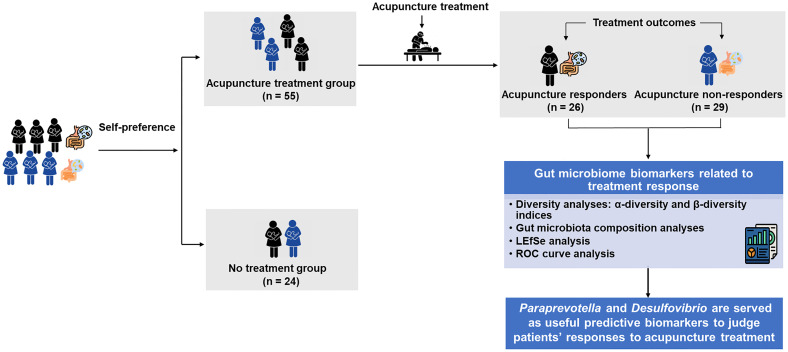

## 1 Introduction

Postpartum depressive disorder (PPD) is a common, disabling, but treatable psychiatric condition (Howard et al., 2014). However, without prompt diagnosis and treatment, maternal suicide and infanticide may be extreme outcomes of PPD (Grigoriadis et al., 2017; Netsi et al., 2018). With a global prevalence of 17.22% (Wang et al., 2021), PPD is a significant maternal and family health burden worldwide. While PPD is most commonly treated with antidepressants and psychological therapies, the efficacy of these approaches varies due to high clinical and functional heterogeneity (Consortium, P.D.A.T.C.a.T.P, 2015; Santos et al., 2017). Indeed, antidepressants have been reported to be only ~42% effective (Brown et al., 2021), and psychotherapy only benefits about a third of PPD patients (Huang et al., 2020). Furthermore, antidepressants have side effects (Carvalho et al., 2016), and any adverse events to the baby during lactation must also be considered (Davanzo et al., 2011). Psychotherapy cannot generally be widely used due to its high cost over long periods of time. An increasing number of PPD patients are seeking safe and effective complementary treatments with few side effects.

Acupuncture is safe and effective in pregnant women (Ormsby et al., 2016; Li et al., 2018; Tong et al., 2019; Li et al., 2020). In a study of 31 meta-analyses and 59 randomized controlled trials, acupuncture was shown to be superior to awaiting treatment, control acupuncture (invasive or non-invasive sham control), and antidepressants in terms of reducing the severity of depression (Hamilton, 1960; Li et al., 2020). Another relatively recent meta-analysis highlighted that acupuncture can significantly reduce Hamilton depression rating (HAMD) scores in PPD patients (Li et al., 2019). However, just like other treatments, the efficacy of acupuncture varies between individuals. PPD therapy urgently requires specific biomarkers to predict therapeutic responses to antidepressant treatments, including acupuncture, so that the correct patients can be prescribed the best treatments at the right time.

Several demographic and clinical therapeutic response predictors to traditional antidepressants in PPD have been reported in robust clinical trials including being white/non-Hispanic (Yonkers et al., 2008), having a major depressive episode within four weeks of delivery (Sharp et al., 2010; Hantsoo et al., 2014), concomitant anxiety symptoms (Cohen et al., 2001), an absence of concomitant psychiatric illness (Yonkers et al., 2008), early response to treatment (Cohen et al., 2001), and improvement within one week after initiation of antidepressants (Appleby et al., 1997). Predictors of non-response included a lifetime history of substance use disorder (Yonkers et al., 2008), concomitant anxiety symptoms (Nonacs et al., 2005), and Hispanic or Black ethnicity (Yonkers et al., 2008). However, the generalizability of these predictors is limited by significant methodological variability including a wide range of studied postpartum periods (2–24 months), comorbid diseases (lifetime alcohol abuse, alcohol dependence, drug abuse, drug dependence, or anxiety disorder) (Nonacs et al., 2005; Suri et al., 2005; Misri et al., 2016), and different severities of depression (minor depression or major depression) (Appleby et al., 1997). In recent years, it has been found that the consistency of quantitative electroencephalographic, the default pattern network with different discrete topological structures in the left and right hemispheres and the variance of the global signal are related to the terminal clinical results of antidepressant treatment of MDD (Hunter et al., 2010; Hou et al., 2016; Zhu et al., 2018), however, the acquisition of the above indicators is undoubtedly complicated, and there are few research results on the efficacy prediction of PPD. Furthermore, predictors focus on clinicodemographic factors and there have been few studies on biomarkers– such as genetic and inflammatory markers (Sharma et al., 2020) – to advance the goal of developing objective and clinically acceptable biomarkers that predict treatment outcomes and guide individualized therapy.

There is now mounting evidence supporting a role for the intestinal microbiota in mental health disorders (Rieder et al., 2017; Dubois et al., 2019; Nikolova et al., 2021; McGuinness et al., 2022). This biochemical signaling pathway, also known as the gut-brain axis, is thought to influence cognitive function and mood via neural, metabolic, hormonal, and immune-mediated mechanisms (Foster and McVey Neufeld, 2013). Previous studies (Chung et al., 2019; Zhou et al., 2020; Nikolova et al., 2021) have found differences in the diversity and composition of gut microbial communities between PPD patients and healthy controls. Changes in the intestinal microflora can affect the efficacy of treatment for some diseases (Ma et al., 2019), and intestinal microflora has recently been shown to be a non-invasive diagnostic biomarker for colorectal adenoma and cancer (Liang et al., 2020). In a systematic review, probiotic therapy showed modest benefits in alleviating depressive symptoms in patients with major depressive disorder over four to nine weeks (Alli et al., 2022). Furthermore, *Lactobacillus rhamnosus* HN001 administered as a probiotic significantly reduced maternal depression and anxiety scores (Slykerman et al., 2017). It is also found that 919 syrup can relieve PPD by regulating the structure and metabolism of intestinal microorganisms and affecting the function of GABA/glutamic acid system in hippocampus (Tian et al., 2021). Additionally, it can be also used to predict responses to cancer immunotherapy in metastatic melanoma patients (Limeta et al., 2020), and dynamic changes in the intestinal microbiota can provide an early prediction of immunotherapy outcomes in patients with hepatocellular carcinoma (Zheng et al., 2019). Recently, some studies have also shown that responses to antipsychotic drugs are related to gut microbiota composition (Schwarz et al., 2018). It is found that the changes of intestinal microbial composition and metabolic function may be related to the response of antidepressants, which provides a potential predictor for the prediction of the curative effect of MDD and can even be used to distinguish MDD from generalized anxiety disorder (Dong et al., 2021; Dong et al., 2022). Therefore, characterizing the nature and impact of the intestinal microbiota on PPD therapy and its value as a biomarker of therapeutic responses would be highly clinically valuable. Acupuncture, as a common complementary alternative therapy, can reduce depressive-like behaviors in chronic unpredictable mild stress (CUMS) rats by regulating intestinal microbes and neurotransmitters (Li et al., 2021). Jiang found that acupuncture can effectively treat all stages of stroke and regulate intestinal flora, thus improving depressive symptoms (Jiang et al., 2023). Therefore, the intestinal microflora may act as clinically relevant biomarkers of therapeutic responses in individuals with mental health diseases, including in those receiving acupuncture.

Here, we first aimed to assess the efficacy of acupuncture in PPD patients. A secondary aim was to identify any differences in the intestinal microbiota in responders and non-responders to acupuncture, with the objective to identify microbiome-based predictors of acupuncture response.

## 2 Materials and methods

### 2.1 Study design

This was a prospective cohort study approved by the Ethics Committee of Shenzhen Hospital of Traditional Chinese Medicine [K2020-027-01]. The study was registered with the Chinese Clinical Trial Registry (http://www.chictr.org.cn/index.aspx; ChiCTR2100041687). Patients with PPD were recruited from the Shenzhen Traditional Chinese Medicine Hospital and Shenzhen Maternity & Child Healthcare Hospital (Shenzhen, China). All procedures used in this study conformed to the ethical standards of national and institutional human experimental committees and the Declaration of Helsinki. All subjects supplied written informed consent (Graphic Abstract).

### 2.2 Participant recruitment

Patients were initially screened for PPD using the Edinburgh Postnatal Depression Scale (EPDS) and then further evaluated using the 17-item Hamilton depression rating scale (HAMD-17) by physicians. All patients were assigned into acupuncture treatment group or no treatment group according to their own preference.

### 2.3 Diagnostic criteria

PPD was diagnosed by the evaluating physician according to the Fifth Edition of the Diagnosis and Statistics of Mental Illness (*DSM-V*) (Battle, 2013; First, 2013). Patients needed to meet five or more of the following symptoms, including at least the first or second symptoms, and the symptoms should have lasted for at least two weeks: (1) low mood and depressive emotion; (2) lack of interest in or loss of enjoyment in activities; (3) significant weight gain or loss; (4) poor sleep, insomnia, or lethargy; (5) psychomotor excitement or retardation; (6) a feeling of fatigue or weakness; (7) a sense that life is worthless, self-accusation, or self-guilt; (8) decline in cognition or difficulty concentrating; and (9) recurrent thoughts of death.

### 2.4 Inclusion and exclusion criteria

The inclusion criteria were: (1) patients between 20 and 49 years of age; (2) a diagnosis of PPD made by a psychiatrist; (3) illness appearing within a year of delivery; (4) HAMD-17 scores between 7 and 24; and (5) providing informed consent, voluntarily participating in the study, and able to complete the assessment instrument.

Exclusion criteria were: (1) severe psychiatric disorders such as bipolar affective disorder and schizophrenia; (2) mental disorder due to brain diseases or for other reasons, and unable to understand the contents of the questionnaire and cannot be effectively evaluated; (3) pregnancy; (4) patients with a HAMD suicide score >2 points; (5) anyone attempting suicide in the past year; and (6) anyone taking antibiotics or probiotics in the past month.

### 2.5 Interventions

Patients in the acupuncture group were treated with acupuncture therapy by an acupuncturist with a doctor’s license and at least three years of clinical experience. Before patient enrollment, all acupuncturists participated in standardized operating procedure training, including locating the acupoints and needle manipulation.

The acupoints selected in this study including Baihui (DU20), Yintang (EX-HN3), Zhongwan (RN12), Qihai (RN6), Guanyuan (RN4), Neiguan (PC6), Shenmen (HT7), Hegu (LI4), Sanyinjiao (SP6) and Taichong (LR3). The location of acupoints has been shown in **Table 1**. When participants were supine, the skin around acupoints were routinely sterilized with 75% alcohol cotton swab, then disposable sterile needles (Product type: HuanQiu, Suzhou, China; 0.3 mm × 40 mm/0.3 mm × 75 mm; C-160630) were inserted into each acupoint to achieve the *deqi* sensation (a sensation of soreness, numbness, swelling, or radioactivity indicating the effectiveness of acupuncture). Paired alligator clips from the electroacupuncture (EA) apparatus (Hwato brand, Suzhou Medical Appliance Factory) were attached transversely to the needle holders at Baihui (DU20) and Yintang (EX-HN3), Zhongwan (RN12) and Qihai (RN6). The EA stimulation lasted for 30 minutes with a continuous wave of 2Hz and a current intensity of 0.1 to 1 mA depending on the participants comfort level. Acupuncture treatment consisted of 16 sessions, each for 30 minutes, and were administered over 8 weeks.

**Table 1:** Localization of acupoints selected in this trial.

Acupoint	Localization
Baihui (DU20)	7 cun above the middle of the posterior hairline, on the middle of the top of the head
Yintang (EX-HN3)	On the forehead, between the brows
Zhongwan (RN12)	On the anterior median line, 4 cun superior to the umbilicus
Qihai (RN6)	On the anterior median line, 1.5 cun caudal to the umbilicus
Guanyuan (RN4)	On the anterior median line, 3 cun caudal to the umbilicus
Neiguan (PC6)	2 cun proximal to the processus styloideus radii, between the tendons of the palmaris longus and the flexor carpi radialis
Shenmen (HT7)	In the wrist, the ulnar end of the transverse striation of the carpal palmar, and the radial depression of the flexor tendon of the ulnar carpal
Hegu (LI4)	On the highest point at m. interosseus dorsalis
Sanyinjiao (SP6)	3 cun proximal to the medial malleolus
Taichong (LR3)	Between metatarsal I and II, just distal to the caput

Patients in the no treatment group didn’t receive any therapy.

### 2.6 Clinical outcomes

The clinical outcome was the response rate. The HAMD is a commonly used scale for clinical evaluation of depressive state (Hamilton, 1960). Depressive symptoms of PPD patients were assessed by HAMD-17 scale (17 items, scored from 0 to 52, higher scores representing more severe the depressive symptoms).

Patients were defined as responders if the HAMD-17 score reduced by ≥50% or the HAMD-17 score was <7 after treatment. Patients were defined as non-responders if the reduction in HAMD-17 score was <50% (Keller, 2003).

### 2.7 Fecal samples collection

Fecal samples of PPD participants in acupuncture treatment group were collected once at baseline and placed in sterile plastic cups, then frozen at −80°C immediately after defecation. The details of fecal sample collection are described elsewhere (Zhou et al., 2019).

### 2.8 DNA extraction and 16S ribosomal RNA gene sequencing

DNA was extracted using the MOBIO PowerSoil^®^ DNA Separation Kit according to the manufacturer’s instructions, and stored at −80° in Tris-EDTA buffer solution before microbial MiSeq sequencing. The V4 region of 16S rRNA gene was amplified by PCR with primers 515F (5’-GTGYCAGCMGCCGCGGTAA-3’) and 806R (5’-GGACTACNVGGGTWTCTAAT-3’), along with barcode sequences, as previously described (Zhou et al., 2020). PCR mixtures contained 1 μl of each forward and reverse primer (10μM), 1 μl of template DNA, 4 μl of dNTPs (2.5mM), 5 μl of 10× EasyPfu Buffer, 1 μl of Easy Pfu DNA Polymerase (2.5 U/μl), and 1 μl of double-distilled water in a 50-μl reaction volume. Thermal cycling consisted of an initial denaturation step at 95° for 5min, followed by 30 cycles of denaturation at 94° for 30 s, annealing at 60° for 30 s, and extension at 72° for 40 s, with a final extension step at 72° for 4min. Amplicons were run for each sample on an agarose gel. Expected band size for 515f-806r was ∼300–350 bp. Amplicons were quantified with Quant-iT PicoGreen dsDNA Assay Kit (P11496; Thermo Fisher Scientific, Waltham, MA, USA) according to manufacturer’s instructions. The amplicon library for high-throughput sequencing on the Illumina MiSeq V3 reagent PE150 (300 cycles) platform was combined to an equal amount and subsequently quantified using KAPA Library Quantification Kit (KK4824; Illumina, Inc., San Diego, CA, USA) according to manufacturer’s protocols.

### 2.9 High-throughput sequencing of 16S ribosomal RNA gene and microbial analysis

High-throughput sequencing analysis was performed using Quantitative Insights into Microbial Ecology (QIIME) 2.0 according to the manufacturer’s instructions. Raw Illumina read data were deposited into tags, reads belonging to each sample were separated with barcodes, and low-quality reads were removed. The processed tags were clustered into amplicon sequence variants (ASVs) using the commonly used 97% similarity threshold. ASVs were assigned to taxa by matching to the SILVA database. A phylogenetic tree of representative sequences was constructed. α-diversity indices such as evenness, observed species, Shannon, and Faith-PD indices were calculated by Wilcoxon rank sum test. For β-diversity indices, firstly, Wilcoxon rank sum test was used to analyze the inter-group and intra-group differences. The former indicated differences in microbial composition between samples within the same group; the later indicate the differences in microbial composition of pairwise samples from different groups. Secondly, the Bray-Curtis dissimilarity and unweighted unifrac calculated by principal coordinate analyses were used for β-diversity indices. To further identify specific bacteria as biomarkers at the genus level, linear discriminant analysis effect size (LEfSe) was applied through the Huttenhower Lab Galaxy Server (Segata et al., 2011) after taxa summaries were reformatted. LEfSe settings were as previously described (Zhou et al., 2020), and systemic forms with a linear discriminant analysis (LDA) cutoff of 2.0 and a *P* < 0.05 in the built-in rank sum test were considered statistically significant. Finally, biomarker data (specific bacteria) calculated by LEfSe were further analyzed by receiver operator characteristic (ROC) curve analysis, and area under the curve (AUC) was used to assess the ROC effect. The cut-off value associated with optimal sensitivity and specificity was used to distinguish acupuncture responders and non-responders.

### 2.10 Analysis of clinical data

The demographic and clinical outcomes were analyzed using the SPSS 22.0 software (IBM Statistics, Armonk, NY, USA). Normally distributed data were analyzed using Student’s *t*-test, while non-parametric data were analyzed using the Mann-Whitney U-test with data expressed as medians with interquartile ranges (IQR). Categorical data were compared using the chi-squared test. A *P*-value < 0.05 was considered statistically significant.

## 3 Results

**Figure 1:**
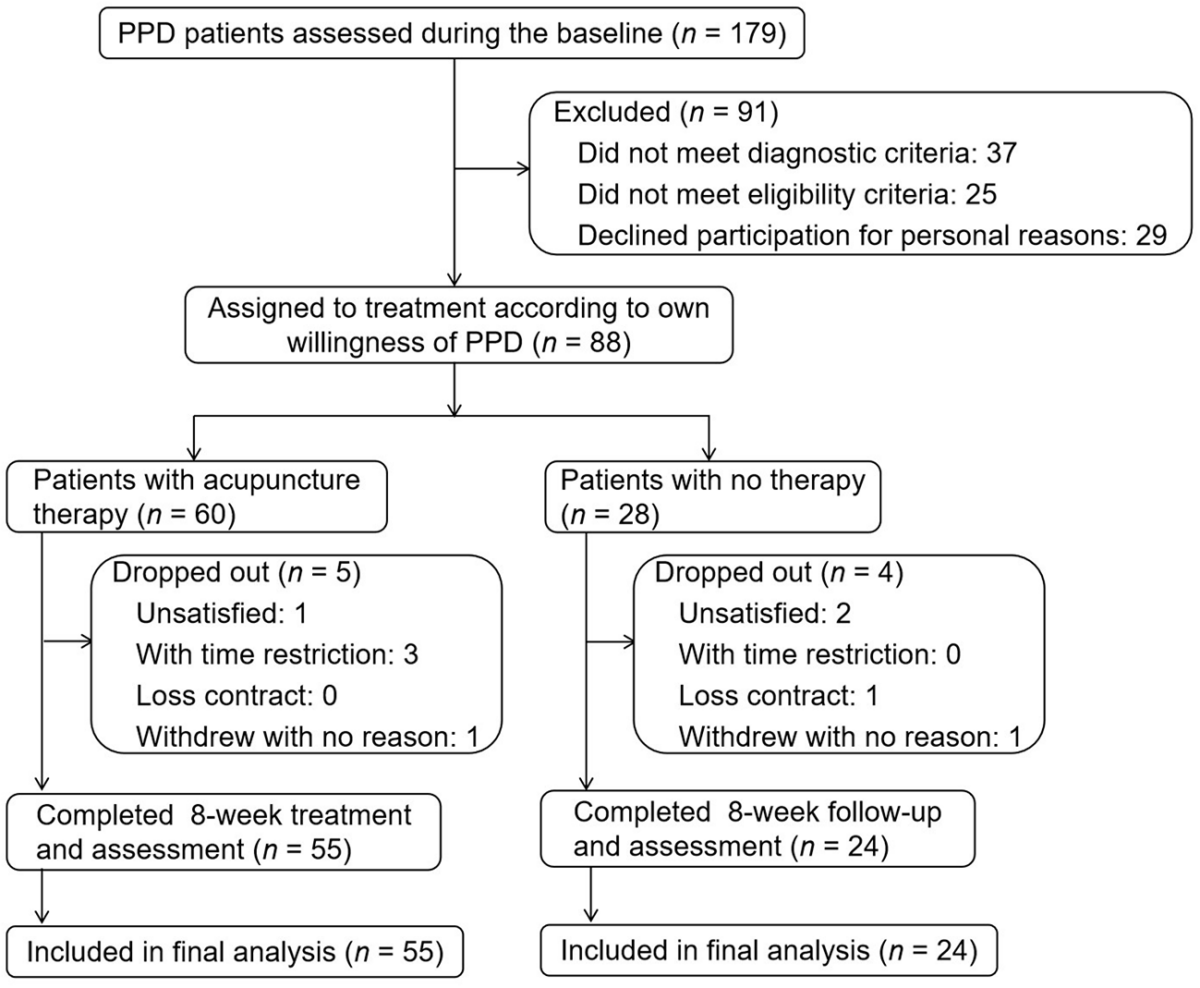
Trial flow diagram.

Among 179 patients screened, 88 were enrolled at baseline between March 25 and November 22, 2021. According to patient preference, 60 received acupuncture treatment (acupuncture group) and 28 received no treatment (control group). During the study, nine (10.23%) patients dropped out: five (8.33%) received acupuncture and four (14.29%) had not received acupuncture. Seventy-nine patients completed the eight-week follow-up and assessments (**Figure 1**).

### 3.1 Effectiveness of acupuncture therapy on PPD

#### 3.1.1 Clinical characteristics of acupuncture therapy and control patients

The baseline demographic and clinical characteristics are shown in **Table 2**. There were no significant differences in age, body mass index (BMI), number of days postpartum, number of parturitions, duration of disease, delivery mode, family history, and EPDS or HAMD-17 scores between those receiving acupuncture and those not receiving acupuncture at baseline.

**Table 2:** Clinical characteristics of patients in the acupuncture therapy and the control groups.

Characteristic	Acupuncture therapy group (N = 55)	Control group (N = 24)	*P*-value
Age (years, mean ± SD)	35.35 ± 4.01	32.21 ± 3.16	0.001
BMI (kg.m^-2^, mean ± SD)	22.56 ± 3.31	21.58 ± 3.71	0.249
Number of days postpartum (days, mean ± SD)	142.24 ± 101.67	125.83 ± 87.60	0.185
Number of parturition(n)	1.53 ± 0.60	1.33 ± 0.57	0.470
Duration of disease (months)	3.78 ± 2.84	3.27 ± 2.52	0.449
Delivery mode (A/B/C)^*^	32/22/1	18/6/0	0.326
Family history (y/n)	4/51	1/23	0.602
EPDS	15.67 ± 4.57	15.71 ± 5.14	0.976
HAMDS	14.00 ± 3.51	13.33 ± 4.78	0.544

*A, Natural childbirth; B, Cesarean section; C, Women miscarried but pregnant for more than seven months.

#### 3.1.2 Comparison of response rates between groups

47.27% responded to acupuncture treatment in the acupuncture group, and 12.5% patients not receiving treatment recovered after 8-week follow up. This difference was significant (*P* = 0.003) (**Table 3**).

**Table 3:** Comparison of response rates between acupuncture group and control group.

	Response N (%)	No response N (%)	Effect size	*P-value*
Acupuncture group (N = 55)	26(47.27%)	29(52.73%)	8.696	0.003
Control group (N = 24)	3(12.5%)	21(87.5%)		

#### 3.1.3 Comparison of HAMD reduction rate between groups

Compared with the HAMD reduction rate between two groups, the results showed reduction rate in acupuncture group was superior than that in control group. This difference was significant (P <0.001) (**Table 4**).

**Table 4:** Comparison of HAMD reduction rate between acupuncture group and control group.

	HAMD reduction rate (%, median (Q1, Q3)^*^)	Effect size	*P-value*
Acupuncture (N = 55)	44.44 (25.00, 64.71)	1126.50	<0.001
Control group (N = 24)	9.19 (-7.55, 20.00)		

^*^Q1, upper quartile; Q3, lower quartile.

#### 3.1.4 HAMD changes between patients receiving acupuncture and controls

Compared with baseline, HAMD scores in the control group did not significantly decrease (*P* = 0.113). However, the HAMD score decreased significantly in patients receiving acupuncture treatment (*P* <0.001) (**Table 5**).

**Table 5:** Comparison of HAMD score before and after treatment in two groups.

Groups	Before treatment	After treatment	Effect size	*P-value*
Acupuncture (N = 55)	14.00± 3.51	7.76 ± 3.83	9.699	<0.001
Control group (N = 24)	13.33 ± 4.80	12.38 ± 4.10	1.647	0.113

### 3.2 Characteristics of the gut microbiota between responders and non-responders before treatment

#### 3.2.1 Baseline clinical characteristics between responders and non-responders

There were no significant differences in age, BMI, number of days postpartum, number of parturitions, length of disease, delivery mode, family history, and EPDS or HAMD-17 scores between responders and non-responders (**Table 6**).

**Table 6:** Clinical characteristics of acupuncture responders and non-responders.

Characteristic	Responders (N = 26)	Non-responders (N = 29)	*P-*value
Age (year, mean ± SD)	34.85 ± 3.96	35.79 ± 4.07	0.386
BMI (kg.m^-2^, mean ± SD)	22.87 ± 3.81	22.27 ± 2.83	0.510
Number of days postpartum (day, mean ± SD)	129.19 ± 91.29	153.93 ± 110.44	0.373
Number of parturitions (n)	1.54 ± 0.58	1.52 ± 0.63	0.898
Duration of disease (months)	3.19 ± 2. 12	4.31 ± 3.31	0.138
Delivery mode (A/B/C)^*^	13/12/1	12/17/0	0.418
Family history (y/n)	1/25	3/26	0.354
EPDS	15.65 ± 3.73	15.69 ± 5.28	0.977
HAMDS	14.42 ± 3.22	13.62 ± 3.77	0.403

*A, Natural childbirth; B, Cesarean section; C, The Women miscarried who are but were pregnant for more than seven months.

#### 3.2.2 Sequencing characteristics

A total of 55 samples from all recruited subjects were sequenced on an Illumina MiSeq sequencer. For downstream analysis, 2259092 qualified reads from 2373462 raw reads were filtered.

#### 3.2.3 Gut microbial diversity changes in acupuncture responders and non-responders

**Figure 2:**
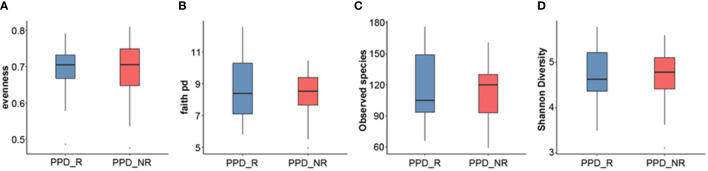
Microbial α-diversity analyses. Evenness index **(A)**, Faith PD **(B)**, observed species **(C)**, Shannon diversity **(D)**. PPD_R, PPD patients who are responsive to acupuncture treatment; PPD_NR, PPD patients who are not responsive to acupuncture treatment.

We next used different diversity indices (evenness, Faith PD, observed species, Shannon diversity) to assess gut microbial α-diversity. There were no significant differences in diversity between acupuncture responders and non-responders (*P* = 0.7856, *P* = 0.4276, *P* = 0.6679, and *P* = 0.7208, respectively). However, the gut microbial diversity, as estimated by evenness, Faith PD, observed species, and Shannon diversity, tended to be higher in responders than non-responders (**Figure 2**).

**Figure 3:**
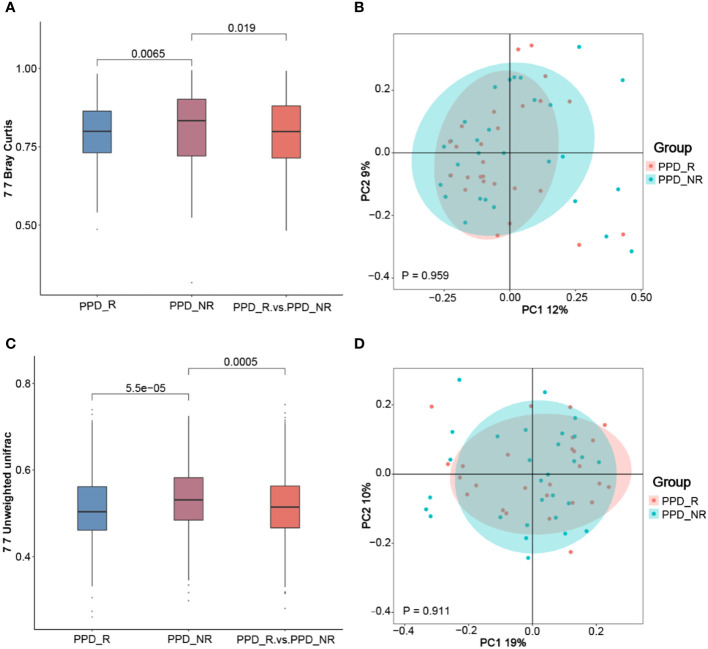
Microbial β-diversity analyses. The Wilcoxon rank-sum test analysis **(A)** and principal coordinates analysis plots **(B)** of the fecal microbiomes based on the Bray-Curtis distance; Wilcoxon rank-sum test analysis **(C)** and principal coordinates analysis plots **(D)** based on the Unweighted-UniFrac distance metric. PPD_R, PPD patients who are responsive to acupuncture treatment; PPD_NR, PPD patients who are not responsive to acupuncture treatment.

To better understand differences in overall community composition between the samples, we calculated Bray-Curtis distances and unweighted UniFrac distances, which were both higher in non-responders than responders (*P* = 0.0065 and *P* = 5.5e^-05^) and between groups (*P* = 0.019 and *P* = 0.0005) (**Figures 3A, C**), To further demonstrate differences in species diversity between samples, we applied the Principal Coordination Analysis and non-metric multidimensional scaling (**Figures 3B, D**). The gut microbial composition was similar between groups, with a tendency to being more centralized in the responder group than in the non-responder group, although this was not statistically significant (P = 0.959 and P = 0.911).

#### 3.2.4 Comparison of gut microbiota composition in acupuncture responders and non-responders

**Figure 4:**
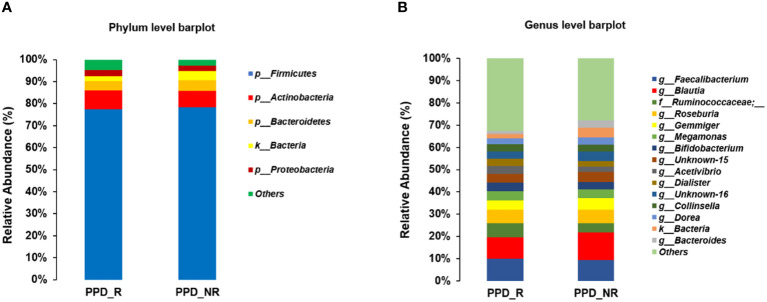
Microbiome composition differences at the phylum **(A)** and genus **(B)** levels between the two groups. PPD_R, PPD patients who are responsive to acupuncture treatment; PPD_NR, PPD patients who are not responsive to acupuncture treatment.

At the phylum level, *Firmicutes, Actinobacteria, Bacteroidetes*, and *Proteobacteria* were the most abundant organisms in the gut microbiota (**Figure 4A**). The genera of *Faecalibacterium, Blautia, Ruminococcaceae, Roseburia, Gemmiger, Megamonas* and *Bifidobacterium* were dominant in the two groups. The 5 genera *Faecalibacterium, Ruminococcaceae, Roseburia, Megamonas* and *Bifidobacterium* had higher abundance in the PPD group (9.99, 6.45, 6.09, 4.04, and 3.96%, respectively) as compared to those in the control group (9.42, 4.08, 6.06, 3.87, and 3.28%, respectively). The 2 genera *Blautia* and *Gemmiger* had lower abundance in the PPD group (9.51 and 4.16%, Gemmiger) as compared to those in the control group (12.37 and 5.41%, respectively). However, the results didn’t reach significance (all *p*-values were more than 0.05) (**Figure 4B**).

#### 3.2.5 Specific genera associated with acupuncture treatment responses

**Figure 5:**
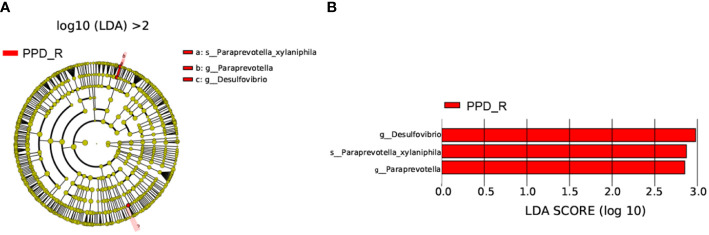
Differences in bacterial taxa between acupuncture responders and non-responders. Cladogram showing the most differentially abundant taxa identified by LEfSe. Red indicates clades enriched in the responder group **(A)**. Comparisons of gut microbiota between acupuncture responders and non-responders **(B)**. Only genera meeting a linear discriminant analysis score threshold >2 are shown. PPD_R, PPD patients who are responsive to acupuncture treatment; PPD_NR, PPD patients who are not responsive to acupuncture treatment.

LEfSe analysis (*p* <0.05, LDA > 2) was used to identify specific bacteria associated with acupuncture treatment responses. *g_Desulfovibrio*, *g_Paraprevotella*, and *Paraprevotella_xylaniphila* were enriched in the responder group (**Figure 5**).

**Figure 6:**
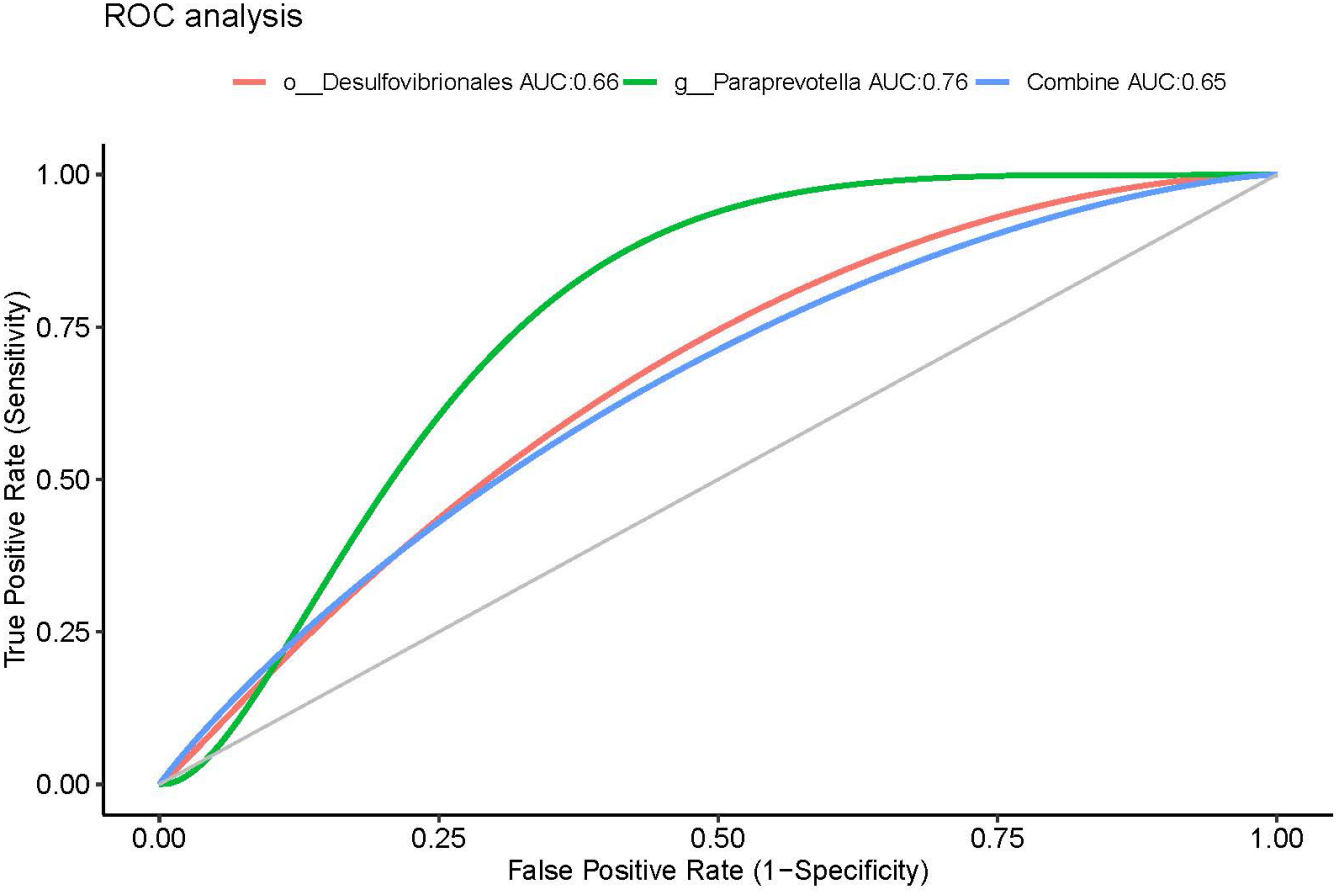
ROC curves using fecal microbiota to distinguish responders from non-responders. AUC, area under the curve; ROC, receiver operating characteristics.

Having identified these three genera (biomarkers), we performed ROC curve analysis to evaluate their predictive accuracy. The area under the curve (AUC) was 0.76 and 0.66 for *g_Paraprevotella* and *g_Desulfovibrio*, respectively. The AUC of combining genera *g_Paraprevotella* and *g_Desulfovibrio* was 0.65 (**Figure 6**).

## 4 Discussion

This trial showed that acupuncture alleviated depressive symptoms in patients with PPD over an 8-week treatment period, and 47.27% patients significantly responded to acupuncture treatment. Additionally, based on gut microbiota profiling, we successfully predicted responses to acupuncture and improvements in clinical symptoms in PPD patients after treatment. *Desulfovibrio* and *Paraprevotella* were identified as specific predictive genera.

So far, there have been few reports on the predictors of therapeutic effect of PPD. Pinna speculated the neurosteroid biosynthesis and endogenous cannabinoid system might be able to predict antidepressive treatment, but lack of rigorous experimental studies to confirm this idea (Pinna, 2023). Additionally, there are also some limited explanations, such as the influence of running on cortisol, which can also affect the therapeutic effect of PPD in the later stage (Gobinath et al., 2018). These prediction methods are often incomplete and indirect. Therefore, gut microbiota as a predictor is seem to be more objective.

In the present study, our results further confirm the close relationship between the gut microbiota and mental health disorders. It has long been known that the enterotype distribution varies according to depression status, with *Bacteroides enteritidis* type 2 more prevalent in depressed patients than healthy controls (Valles-Colomer et al., 2019). It has also been shown that intestinal microbiota disorder is a characteristic of major depressive disorder (MDD) patients (Zheng et al., 2016; Zheng et al., 2020). Duan et al. studied treatment responses to escitalopram in a CUMS mouse depression model, comparing changes in metabolic function before and after treatment, and found that treatment responses were related to microbial composition, providing new insights into the mechanisms underlying variable antidepressant efficacy (Duan et al., 2021). Therefore, the flora structure is closely related to the intrinsic pathobiology of depression, suggesting that intestinal microbial biomarkers may be good predictors of antidepressant treatment responses.

Most studies employ a multifaceted approach to characterizing the gut microbiota, usually including measures of both α- and β-diversity. α-diversity is commonly used as a surrogate of community stability and function, which are thought to be beneficial to the host (Shade, 2017). Jiang et al. found that the intestinal microflora α-diversity was higher in antidepressant drug non-responders than responders in MDD patients compared with healthy controls (Jiang et al., 2015). In addition, the α-diversity of the gut microbiota was not significantly different in MDD patients with different treatment responses (Dong et al., 2022). In our study, we found that there were no significant differences in diversity between PPD patients who did and did not response to acupuncture, nor were there differences in the abundance and uniformity of the gut microbiota between the two groups. This mirrors the inconsistent results of previous studies, and the specific reasons underlying these differences need further study.

β-diversity reflects relationships between samples by analyzing the species composition and abundance (Anderson et al., 2011). Our β-diversity analysis showed that responders were significantly separated from non-responders, and the responder group had a more similar species composition. Kelly et al. and Zheng et al. both reported significant differences in β-diversity between individuals with depression and healthy controls (Kelly et al., 2016; Zheng et al., 2016). In the CUMS-induced depression study in mice, the β-diversity was also different between non-responders and responders (Duan et al., 2021), as was the β-diversity in patients who did and did not benefit from anti-programmed cell death protein 1 (PD-1) immunotherapy (Mao et al., 2021). The latter study found that the intestinal microflora affected the spectrum of immunotherapy-related adverse events, with high species diversity and relative abundance perhaps protective against immunotherapy-related adverse events (Mao et al., 2021).

We further analyzed and identified specific genera associated with acupuncture treatment responses. At the genus level, *Desulfovibrio* and *Paraperevotella* were enriched in responders, consistent with previous studies reporting a high abundance of *Paraprevotella* and *Desulfovibrio* at the genus level in patients with depression (Naseribafrouei et al., 2014; Chen et al., 2018a; Chen et al., 2018b). *Desulfovibrio* are present in the oral and intestinal tract of approximately 50% of people, where they release hydrogen sulfide as a product of sulfate reduction (Devereux et al., 1990). Hydrogen sulfide is involved in the natural prevention of many digestive tract diseases (Pires et al., 2006), and there is a well-established link between desulfurization bacteria and individual intestinal diseases (Verstreken et al., 2012). For example, Scanlan et al. found significantly more desulfurization bacteria in the feces of colon cancer patients than healthy people (Scanlan et al., 2009), and similarly Rowan et al. found a significantly higher relative abundance of desulfurization bacteria in the intestinal tracts of patients with ulcerative colitis than those of healthy controls (Rowan et al., 2010). Additionally, we discovered that *Paraprevotella* was enriched in the gut microbiota of patients responding well to acupuncture. *Paraprevotella* belongs to the *Prevotellaceae* family, and another family member *Prevotella* is associated with a healthy plant-based diet and probiotic use (Ley, 2016). *Prevotella* can also act as an opportunistic pathogen associated with periodontal and dental inflammation, intestinal inflammation, rheumatoid arthritis, and bacterial vaginitis (Arweiler and Netuschil, 2016; Randis and Ratner, 2019; Bertelsen et al., 2021; Jia et al., 2021).

*Desulfovibrio* and *Paraperevotella* have different potential pathogenic mechanisms. For example, *Desulfovibrio* organisms co-cultured with human oral epidermoid carcinoma (KB) cells increased interleukin (IL)-6 production, implicating them in immune responses (Bisson-Boutelliez et al., 2010). Colonization of the intestine with *Prevotella* leads to metabolic changes in the microbiota that reduce IL-18 production (Iljazovic et al., 2021), thus aggravating intestinal inflammation and possibly leading to systemic autoimmunity. Furthermore, *Prevotella* can damage intestinal mucosal barrier function by producing sulfatase, which induces and degrades mucus, thus helping itself and other harmful bacteria to access intestinal epithelial cells to generate local inflammation (Wright et al., 2000). In addition, these two genera as Gram-negative bacteria might help to explain the role of microbiota in the development/maintenance of depression. Gram-negative bacteria contain lipopolysaccharides in the outer cell membrane leaflet (Al Bander et al., 2020), and lipopolysaccharides interacts with macrophages and stimulates immune responses through pro-inflammatory cytokine release. Supporting this, increased levels of proinflammatory cytokines including IL-1β and IL-6 and decreased levels of anti-inflammatory cytokines including IL-4 and IL-10 have been detected in people living with depression (Berk et al., 2013; Wong et al., 2016).

An increasing number of studies show that the occurrence and development of depression are closely related to inflammation and immunity (Simmons and Broderick, 2005; Maes et al., 2012; Kelly et al., 2015), Inflammatory cytokines and kynurenine pathway have been found as potential therapeutic targets for PPD, because the increase of plasma IL-6 and IL-8 and the decrease of serotonin, IL-2 and quinolinic acid are related to the severity of depressive symptoms, which increases the risk of PPD (Achtyes et al., 2020). These results indicate that the increased level of some inflammatory biomarkers in PPD patients means that the disease is related to the impaired adaptability of the immune system (Bränn et al., 2020).

Therefore, the high expression of these genera in PPD patients may correspond to increased levels of inflammatory biomarkers, and several studies have shown a strong association between persistent inflammatory responses and antidepressant therapy resistance (Carvalho et al., 2013). Electroacupuncture can downregulate inflammatory factors such as IL-6 in the hippocampus of depressed rats, suggesting that electroacupuncture may relieve depression through immune regulation (Guo et al., 2014; Yue et al., 2018). Indeed, α7nAChR is activated by acetylcholine released from cholinergic nerve endings and is a key target for inhibiting pro-inflammatory cytokines release by macrophages (Stakenborg et al., 2017). Acupuncture can reduce inflammatory cytokine production through the vagus nerve by activating α7nAChR (Yang et al., 2021). Acupuncture can also regulate the interaction between the gut microbiota and the brain-gut axis, inhibit proinflammatory cytokine production, alter the number and proportion of the gut microbiota, restore its stability, improve intestinal barrier function, and further adjust body function (Jang et al., 2020; Wang et al., 2020). In acupuncture treatment of PPD, the unique regulation mechanism of immune intestinal flora also played an important role. Finally, we found that acupuncture might inhibit inflammation and improve depression via two pathways: (1) inhibiting the release of inflammatory cytokines by activating the vagus nerve; and (2) regulating the brain-gut axis through the intestinal microflora, some predecessors put forward the same argument previously. (Yang et al., 2022).Therefore, accumulation of *Desulfovibrio* and *Paraperevotella* in the intestinal tracts of responsive patients could mediate the immune response induced by acupuncture to better regulate and alleviate depressive symptoms. The conclusion of our research results accords with the above conclusion, which can be understood as that acupuncture has played a better and more sensitive role in the flora of the responders.

## 5 Limitations

The study has several limitations. The sample size of present study was relatively small, and further studies in larger sample sizes are needed to confirm the findings with more advanced analyses methods, such as machine learning methods.

## 6 Conclusion

In conclusion, *Paraprevotella* and *Desulfovibrio* predicted early responses to antidepressants in patients with PPD receiving acupuncture. These results may help clinicians optimize their management of individual PPD patients in the future. Baseline enrichment and metabolism of *Paraprevotella* and *Desulfovibrio* intestinal microbiota in PPD patients were related to treatment outcomes. These findings pave the way for a new approach to personalize and maximize the efficacy of acupuncture treatment in PPD patients and provide potential new and accurate biomarkers for managing PPD patients.

## Data availability statement

The original contributions presented in the study are publicly available. This data can be found here: https://www.ncbi.nlm.nih.gov/, with accession number PRJNA976190.

## Ethics statement

The studies involving humans were approved by Ethics Committee of Shenzhen Traditional Chinese Medicine Hospital. The studies were conducted in accordance with the local legislation and institutional requirements. The participants provided their written informed consent to participate in this study.

## Author contributions

Y-MZ and J-JY contributed equally. M-P and Z-XY are the corresponding authors. Y-MZ conceived and planned the experiments. Y-MZ and J-JY wrote the manuscript. Y-QX, X-MM, YYXZ, Y-HG, CC and X-XH executed the experiments. M-P and Z-XY contributed to revise the final manuscript. All authors contributed to the article and approved the submitted version.

## Glossary

**Table:**

anti-PD-1	anti-programmed cell death protein 1
AUC	area under the curve
CUMS	chronic unpredictable mild stress
DSM-V	Fifth edition of the Diagnostic and Statistical Manual of Mental Disorders
EPDS	Edinburgh Postnatal Depression Scale
HAMD	Hamilton Depression Rating
HAMD-17	17-item Hamilton Depression Rating
IL	interleukin
LDA	linear discriminant analysis
LEFSe	linear discriminant analysis effect size
MDD	major depressive disorder
PPD	postpartum depressive disorder
PPD_R	postpartum depressive disorder patients who are responsive to acupuncture treatment
PPD_NR	postpartum depressive disorder patients who are not responsive to acupuncture treatment
ROC	receiver operating characteristic curve
rRNA	16S ribosomal RNA
